# Case report: Transmuscular migration of a solid encircling silicone band through all four rectus muscles

**DOI:** 10.3389/fmed.2024.1511566

**Published:** 2025-01-07

**Authors:** Ruiping Gu, Kaicheng Wu, Chunhui Jiang

**Affiliations:** ^1^Department of Ophthalmology and Vision Science, Eye and ENT Hospital of Fudan University, Shanghai, China; ^2^Shanghai Key Laboratory of Visual Impairment and Restoration, Fudan University, Shanghai, China; ^3^NHC Key Laboratory of Myopia (Fudan University), Laboratory of Myopia, Chinese Academy of Medical Sciences, Shanghai, China

**Keywords:** scleral buckling surgery, transmuscular migration, rhegmatogenous retinal detachment, cheese wire forward, encircling band

## Abstract

**Purpose:**

We present a case of transmuscular migration of a solid encircling silicone band through all four rectus muscles.

**Observations:**

A 33-year-old male with high myopia presented with a progressively worsening subclinical peripheral rhegmatogenous retinal detachment in his left eye. An encircling silicone band (#240) was placed anterior to the equator, and 5-0 polyester sutures (Ethicon, Inc.) were used to secure the band at all four quadrants. Six months later, the encircling band and suture knots were visible under the bulb conjunctiva, close to the limbus in all four quadrants. One and a half years after the retinal detachment surgery, the encircling band was removed because of a mild foreign body sensation. During that surgery, it was noted that the encircling band was located anterior to the insertion of all four rectus muscles. Throughout the entire follow-up period, the position and movement of the left eye remained normal.

**Conclusion and importance:**

A deep and long bite of the sclera should be used to secure the silicone band, especially in cases of high myopia and when the band is placed anterior to the equator. Even if the encircling silicone band cheese wire through the four rectus muscles, ocular motility may still remain normal.

## Background

The migration of extrascleral explants is an uncommon complication following the buckling procedure. Lanigan first reported the anterior migration of solid silicone circumferential explants in five patients in 1992 (1). Since then, several authors have reported similar cases (2–5). While migration of the encircling silicone band through one rectus muscle is rare, transmuscular migration through all four rectus muscles is extremely rare (6). Here, we describe a case of anterior migration of an encircling silicone band through all four rectus muscles. A deep and long bite of the sclera should be used to secure the silicone band, especially in highly myopic cases and when the band is placed anterior to the equator. Even if the encircling silicone band cheese wire through the four rectus muscles, ocular motility may still remain normal.

## Case presentation

**Figure 1:**
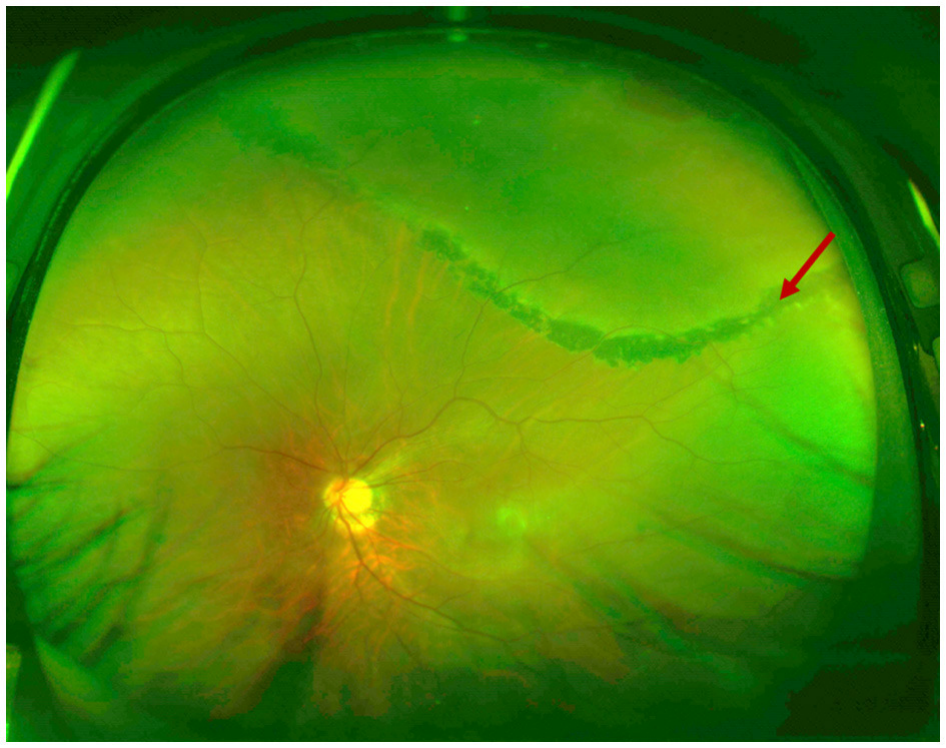
Ultra-wide field retinal images of the rhegmatogenous retinal detachment. The retinal detachment extended beyond the laser mark and progressed further. Red arrow: laser mark.

**Figure 2:**
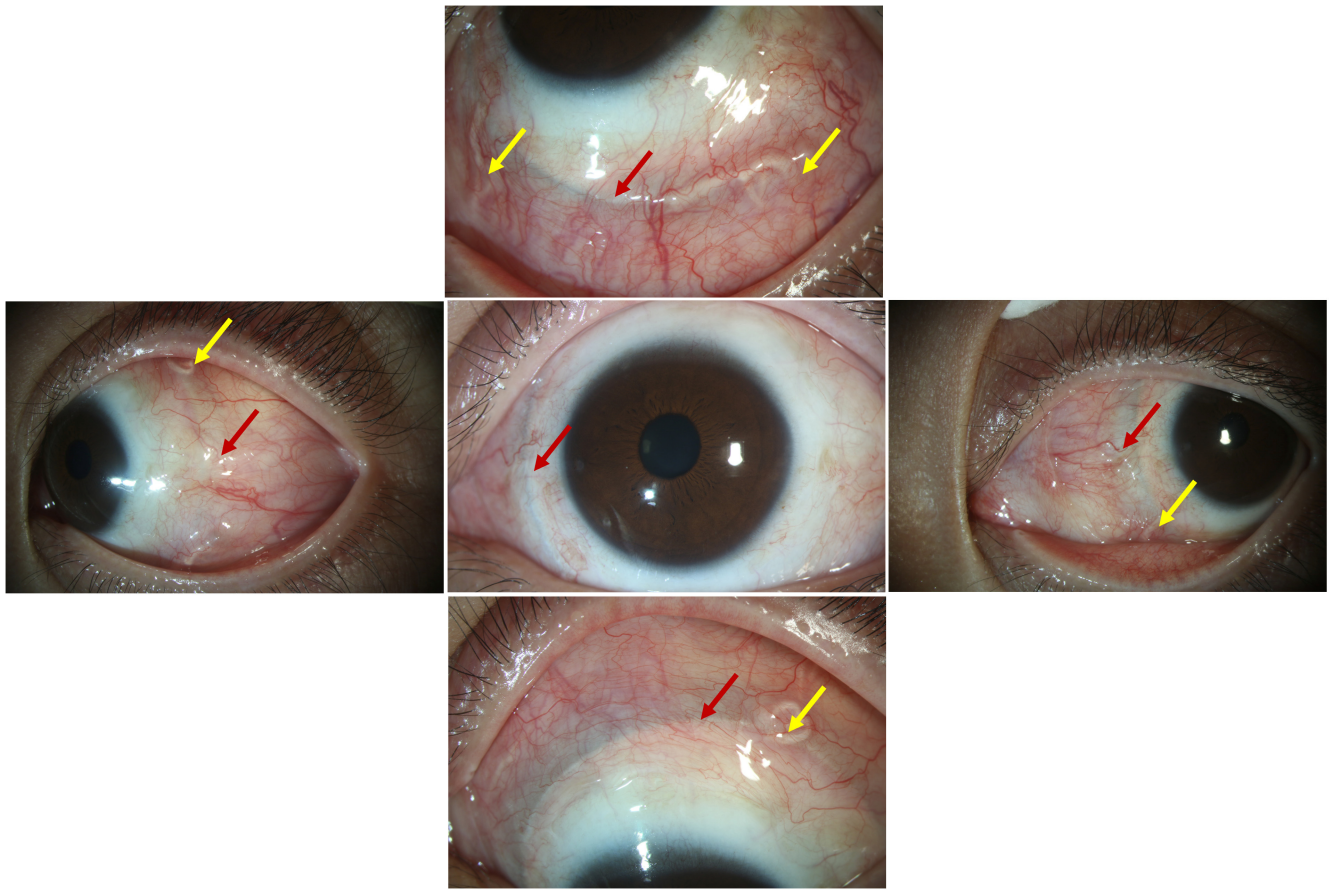
The encircling band and suture knots were visible under the bulb conjunctiva at all four quadrants near the limbus; red arrow: the encircling band; yellow arrow: the suture knots.

**Figure 3:**
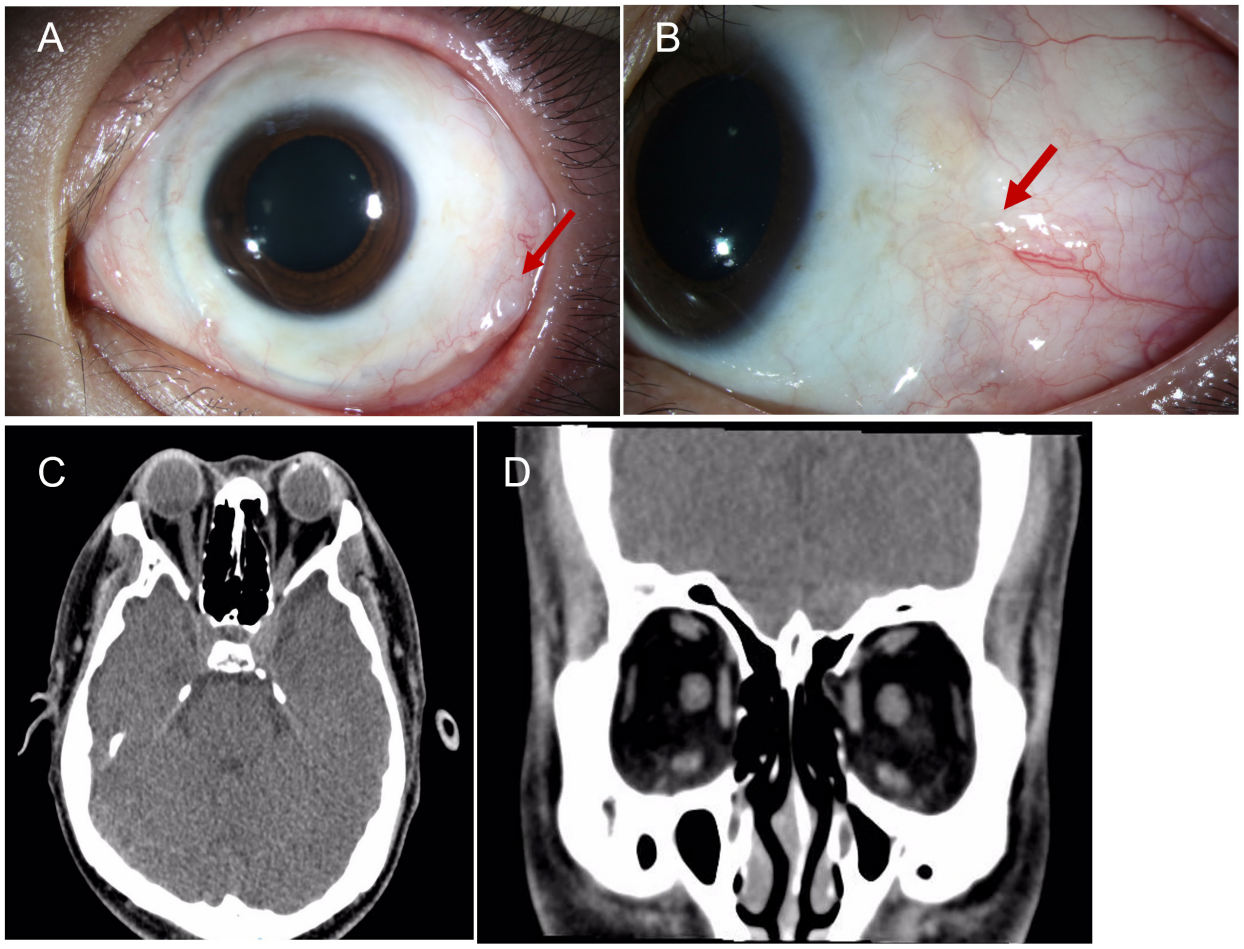
**(A, B)** A severe granuloma was found in the temporal bulb conjunctiva around the band. **(C, D)** The orbital CT showed fractures of the orbital medial wall without entrapment of the internal rectus muscle on the left side, and the four rectus muscles of both eyes were intact; red arrow: granuloma.

**Figure 4:**
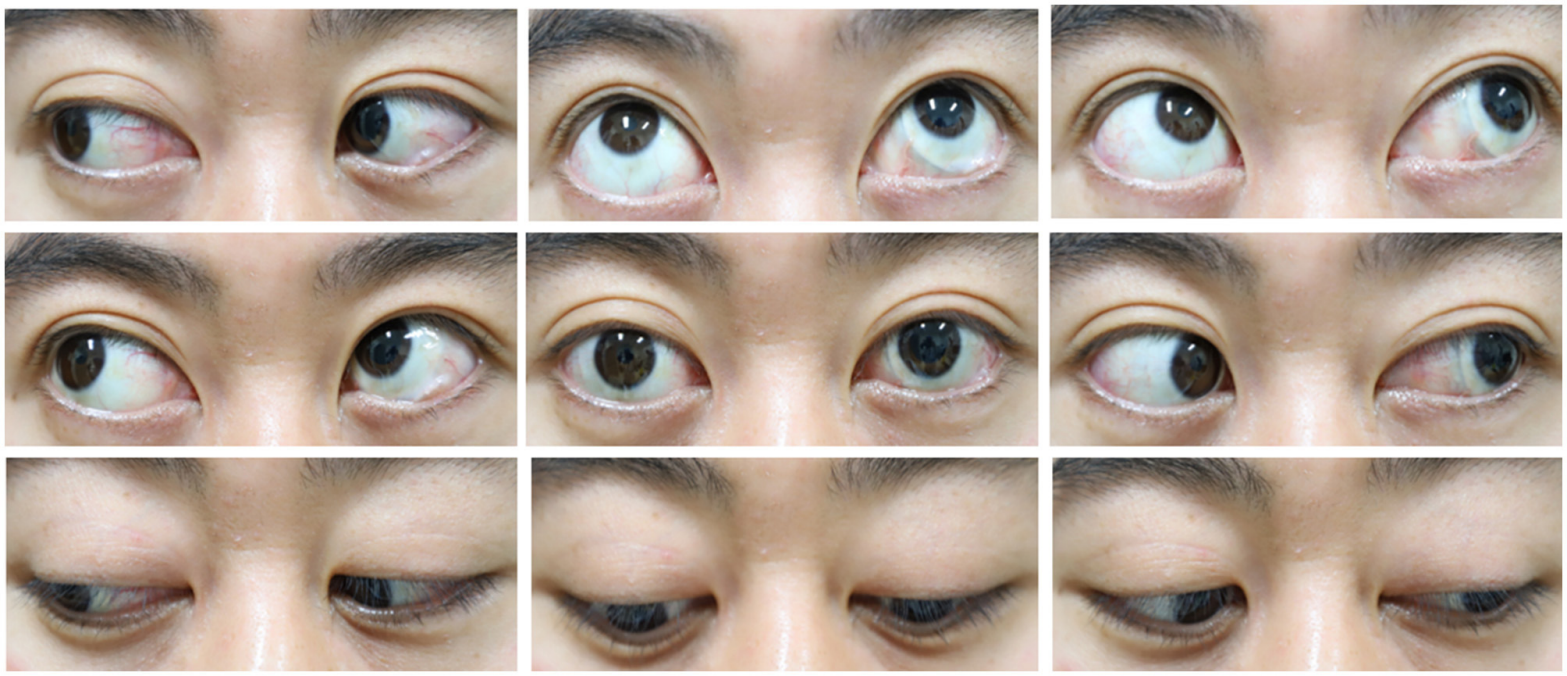
The movement of both eyes was normal.

A 33-year-old male presented with a subclinical peripheral rhegmatogenous retinal detachment in his left eye, which was treated with retinal photocoagulation. The patient also had a history of orbital trauma 10 years ago. Six months later, the retinal detachment in the left eye extended beyond the laser mark and progressed further (Figure 1). An encircling silicone band (#240) was placed anterior to the equator, and 5-0 Polyester sutures (Ethicon, Inc.) were used to secure the band at all four quadrants (one suture in each quadrant). The retina was successfully reattached. Another 6 months later, the encircling band and suture knots were visible under the bulb conjunctiva, close to the limbus in all four quadrants (Figure 2). The patient did not report any discomfort or diplopia. The position and movement of the eye remained normal. One and a half years after the retinal detachment surgery, the patient complained of a mild foreign body sensation in the left eye. Upon examination, in addition to the migration of the silicone band, a severe granuloma was found in the temporal bulb conjunctiva surrounding the band (Figures 3A, B). The orbital computed tomography (CT) showed fractures of the orbital medial wall without entrapment of the internal rectus muscle in the left orbit. The four rectus muscles of both eyes were intact, but the internal rectus muscle of the left eye was slightly thickened (Figures 3C, D). The position and movement of both eyes remained normal (Figure 4). The encircling band was then successfully removed. During that surgery, it was noted that the encircling band was located anterior to the insertion of the four rectus muscles. At the patient's most recent follow-up, his retina remained well attached, and the position and movement of the left eye were still normal. The patient was very satisfied with the results of the treatment.

## Discussion and conclusion

Here, we report a case of a silicone band cheese wiring through all four rectus muscles without causing significant issues with either the position or movement of the eye. No symptoms of anterior segment ischemia were found in this patient.

Lanigan first reported the anterior migration of extrascleral explants in five cases following extrascleral buckling procedures and suggested that the anteriorly directed pressure from the advancing edge of the explant may cause local tissue necrosis and/or ischemia, eventually leading to “cheese wiring” through the muscle insertion (1). Since then, the speculation of cheese wiring through the rectus muscle postoperatively has been promoted (2–4). The extraocular muscles might form adhesions with the implanted material and thus remain firmly attached to the globe despite the erosion of the buckle through the muscle insertions. There is sufficient time for the muscle fibers or sheath to reattach spontaneously and closely to their original insertion (1–4).

In Yui Nishida's case, the encircling band was fixed in the scleral tunnel at all four quadrants, and band migration possibly resulted from the scleral flaps being thin (5). Lanigan reported that the non-absorbable anchoring sutures either cut out or were not visible (1). A torn scleral suture was seen tied to the band in Isaac Ashkenazi's case (2). In our case, the suture knots at three quadrants remained anchored to the silicone band and had shifted away from their original sutured positions. This indicates that the band either left its fixation site with or without the suture first and then cheese wired through the rectus muscle. Therefore, insufficient fixation to the sclera might have contributed to these cases, including ours. The axial length of our case was 26.65 mm, and the sclera was found to be thin in the highly myopic eyes. In addition, the intraoperative cryocoagulation might have further impaired the tissue. During the surgery, we found that the silicone band, along with the sutures, migrated anteriorly. On the other hand, the retinal hole was not too far behind the ora serrata, so the silicone band was placed anterior to the equator. The risk factors for the migration of the silicone band have been identified as placement of the band anterior to the equator, excessive tightening of the band, or insufficient anchoring of the band to the sclera (1–5). Our case exhibited two of these factors.

Interestingly, in our case, although the band migrated through all four rectus muscles, the mobility of the eye was not impaired and there were no signs of anterior ischemia. Diplopia and ocular motility disturbances resulting from migrating buckling elements through the rectus muscles have been well documented (1, 2, 6). In Lanigan's case series of five patients with solid silicone circumferential explant transmuscular anterior migration, two had ocular motility disturbances. Thus, transmuscular migration of the explant did not necessarily lead to ocular motility disturbances. In three of the five patients in Lanigan's case series, no diplopia was found (1). Yui Nishida reported a 58-year-old man with an encircling band exposed on the nasal side of the conjunctiva near the limbus, without any symptoms (5). In our present case, the 240-slicone encircling band cheese wired through all four rectus muscles without causing diplopia. These findings may indicate that the muscle fibers or sheath reattached relatively close to their original location.

Lanigan found prominent episcleral anterior ciliary collaterals from the neighboring rectus muscle circulations, which apparently anastomosed with the cheese wire rectus circulation (1). In our present case, all four rectus insertions were cut and no collateral circulation was found. The reason for this is unclear. Here, the four rectus muscles might not have been cut simultaneously and new circulation might have already been established in one rectus muscle before the band cheese wired through another. This is supported by studies indicating that surgery on all four rectus muscles in healthy patients is safe when performed in a staged fashion (7, 8).

In conclusion, a deep and long bite of the sclera should be used to secure the silicone band, especially in highly myopic cases or when the band is placed anterior to the equator. Even if the encircling silicone band cheese wire through the four rectus muscles, the ocular motility may still remain normal.

## Data Availability

The original contributions presented in the study are included in the article/supplementary material, further inquiries can be directed to the corresponding author.
